# Mechanistic and Translational Advances Using iPSC-Derived Blood Cells

**DOI:** 10.33696/pathology.1.010

**Published:** 2020

**Authors:** Christopher S Thom, Stella T Chou, Deborah L French

**Affiliations:** 1 Division of Neonatology, Children’s Hospital of Philadelphia, Philadelphia, PA, USA; 2 Division of Hematology, Children’s Hospital of Philadelphia, Philadelphia, PA, USA; 3 Center for Cellular and Molecular Therapeutics, Children’s Hospital of Philadelphia, Philadelphia, PA, USA

**Keywords:** iPSC, Hematopoiesis, Developmental biology, Anemia, Thrombosis, Immunodeficiency, Cancer

## Abstract

Human induced pluripotent stem cell (iPSC)-based model systems can be used to produce blood cells for the study of both hematologic and non-hematologic disorders. This commentary discusses recent advances that have utilized iPSC-derived red blood cells, megakaryocytes, myeloid cells, and lymphoid cells to model hematopoietic disorders. In addition, we review recent studies that have defined how microglial cells differentiated from iPSC-derived monocytes impact neurodegenerative disease. Related translational insights highlight the utility of iPSC models for studying pathologic anemia, bleeding, thrombosis, autoimmunity, immunodeficiency, blood cancers, and neurodegenerative disease such as Alzheimer’s.

## Background

The human induced pluripotent stem cell (iPSC) technology was developed more than ten years ago and has provided important tools for the mechanistic and cellular interrogation of many diseases [1]. To generate iPSC lines, somatic cells are reprogrammed into cells resembling embryonic stem cells by the overexpression of key transcription factors. The power of the iPSC technology is that these cells have unlimited regenerative potential, and theoretically have the ability to differentiate into all human cell and tissue types while retaining their genetic identity.

Hematopoiesis is one of the best characterized developmental systems, given relatively accessible patient samples, and excellent cell and animal models [2,3]. Population studies have identified thousands of genomic loci that affect human blood traits [4], leading to translationally relevant developmental insights (e.g., [5–10]). Mechanistic studies using cells derived from clinically affected patients also offer increasingly powerful approaches to discover genetic factors that regulate hematopoiesis, and to identify novel treatment strategies. Hematologic diseases present significant health burdens globally, causing pathologic anemia, bleeding, thrombosis, immune dysfunction, and malignancies. For example, thromboembolic events account for 1 in 4 deaths worldwide [11]. Anemia, with associated morbidities, afflicts ~30% of the global population [12].

**Figure 1:**
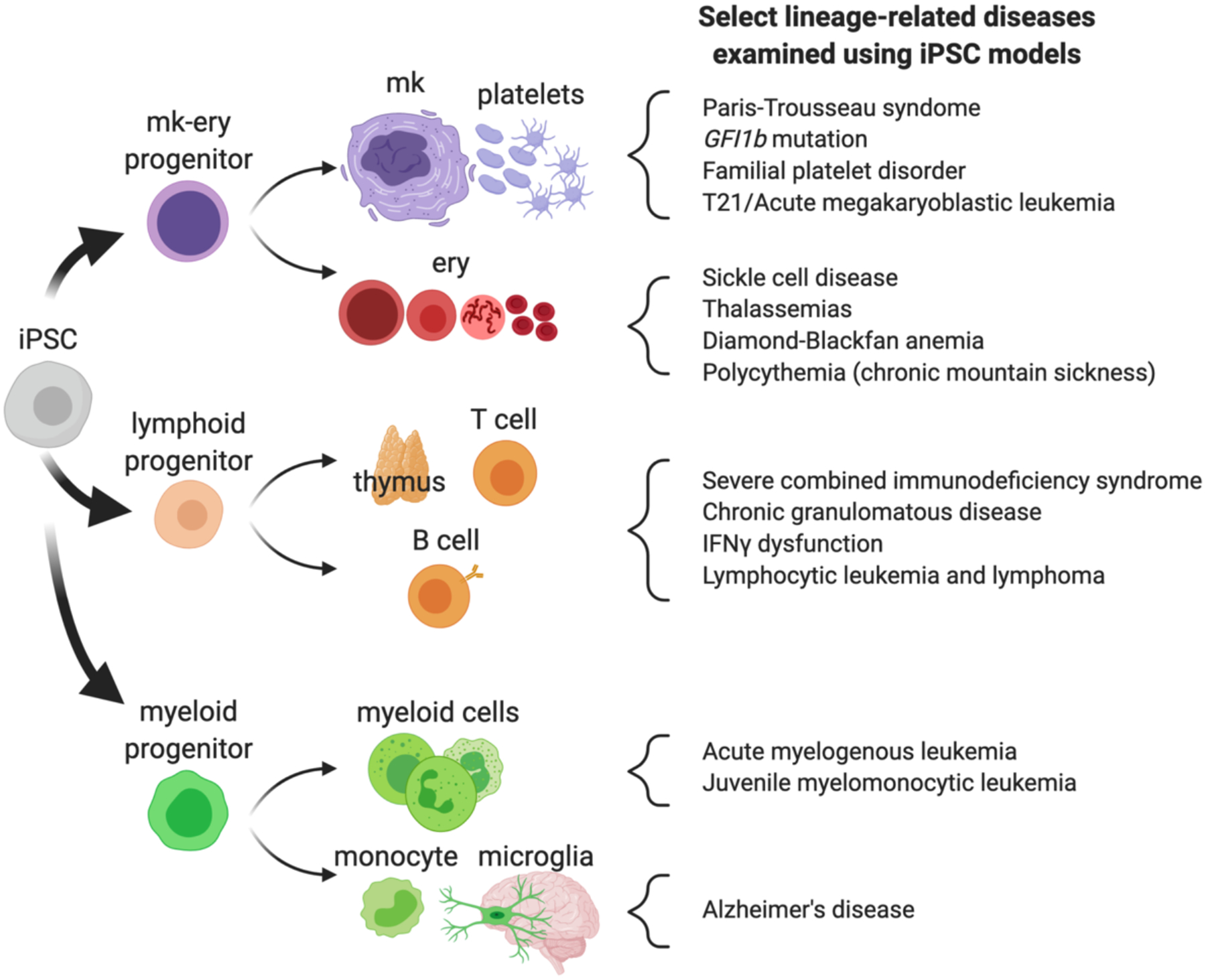
Hematopoietic development schematic and blood cell-related disorders discussed in this article. mk: Megakaryocyte; ery: Erythroid (red blood cell lineage). Created using BioRender.com.

Human iPSCs have been used to model an expanding repertoire of blood development stages and cell types [13,14], including many genetically heritable blood disorders (Figure 1). Given some species-specific differences in hematopoiesis [15], iPSCs represent uniquely suitable reagents for understanding disease pathology and/or treatment.

Blood cells, including tissue-resident macrophages, also play roles in non-hematopoietic organogenesis and disease pathology [16,17]. For example, monocyte-derived microglial cells, derived from embryonic hematopoietic stem cells, impact neurodegenerative diseases. Human iPSC model systems have helped to clarify important roles for these hematopoietic cells in neural disease pathology.

This commentary will focus on studies that have used human iPSC-derived blood cells to elucidate i) mechanisms and treatments for blood disorders, and ii) mechanisms by which blood cells contribute to neurodegenerative disease pathology. For other systems not discussed, the reader is referred to excellent reviews [13,14,18]. Selected studies demonstrate the power of the human iPSC technology to define mechanisms that regulate blood cell development, modeling hematopoietic diseases, and as a platform for novel therapeutic development.

## Ontogeny of Hematopoiesis and Its Relation to iPSC-derived Model Systems

Hematopoiesis describes the process through which stem cells produce all blood lineages. The ontogeny of hematopoiesis involves three separate ‘waves’ of blood development [19]. The first two waves occur within the embryonic yolk sac and are designated primitive and definitive-like hematopoiesis. The third wave occurs later in development in the aorta-gonad-mesonephros region of the embryo and is designated definitive hematopoiesis.

Each wave produces different cell lineages and/or types of terminally differentiated cells. *In vivo*, the 1^st^ primitive wave generates red blood cells expressing embryonic hemoglobin, megakaryocytes/platelets, and myeloid cells. The 2^nd^ definitive-like wave includes red blood cells expressing fetal hemoglobin, in addition to cells of the megakaryocyte, myeloid, and lymphoid lineages. The 3^rd^ definitive wave produces the hematopoietic stem cells (HSCs) that seed the fetal liver and bone marrow to ultimately yield adult-type red blood cells expressing adult hemoglobin, megakaryocyte, myeloid and lymphoid cells throughout life. In addition to the hematopoietic system, blood cells contribute to development and complex disease pathology in virtually all other organ systems [20]. For example, tissue-resident macrophages help to orchestrate tissue development, homeostasis, and repair throughout the body [16,17]. In addition to autoimmune and cancer risks, hematopoietic cell types impact neuropsychiatric and cardiovascular disease [20].

Directed iPSC differentiation protocols that recapitulate many of the signals that occur in the embryo have modeled primitive and definitive-like blood cell lineage development [21,22]. Limitations to these model systems still exist, such as robust generation of enucleated red blood cells, polyploid megakaryocytes, and lymphoid cell lineages. Recent protocols for definitive-like hematopoiesis have addressed some of these limitations [23,24] and many groups are working on protocols to identify the hematopoietic stem cell that would be transplantable and from which definitive cells arise.

## Exemplary Uses for iPSC-derived Red Blood Cells and Related Disease Models

Hereditary anemias arise from defects in hemoglobin, enzyme production, or membrane proteins that adversely impact erythroid maturation or function. Affected patients can have life-long transfusion-dependence, prompting research efforts aimed at producing erythrocytes at clinical scale for transfusion and/or for red cell antibody screening and identification [25–27]. Human iPSCs can model many aspects of erythropoiesis (Figure 1) [28]. Typical assays monitor induction of erythroid gene expression, hemoglobin accumulation, and morphologic changes during erythroid maturation. To a limited extent, iPSC-derived erythrocytes can also extrude their nuclei to form anucleate reticulocytes [25].

Patient-derived iPSCs have been used to define how pathologic erythropoiesis occurs in chronic mountain sickness (CMS, Monge’s disease). Affected individuals living at high altitude can have severe polycythemia, or high red blood cell counts, resulting in myocardial infarction and stroke [29]. A genetic screen identified *SENP1* polymorphisms in Andean individuals with chronic mountain sickness versus individuals living at high altitude without this disorder. The use of iPSC lines from affected and unaffected individuals showed that *SENP1* played a fundamental role in hypoxia-induced polycythemia. When cultured in hypoxic conditions (5% O_2_), cells from affected individuals showed higher SENP1 expression and a ~100-fold increase in CD235a^+^ erythroid cells versus cells from individuals residing at high altitude without CMS. Increased SENP1-mediated desumoylation and activation of GATA1, as well as anti-apoptotic BCL-xL, were responsible for these effects [29]. These findings helped mechanistically define the increased erythropoietic drive underlying the clinical phenotype.

Patient-derived iPSCs have provided proof-of-principal for beta globin gene correction [30,31], paving the way for ongoing clinical trials for thalassemia and sickle cell disease (SCD) [32,33]. Allogeneic hematopoietic stem cell transplant is already a first-line treatment to cure SCD and thalassemia in some parts of the world [34], but autologous transplant of gene-corrected cells would eliminate risks of graft versus host disease and graft rejection.

Human iPSCs have also been used as chemical screening platforms for novel erythroid disease therapies. For example, Diamond-Blackfan anemia (DBA) causes defective erythropoiesis due to genetic perturbations in ribosomal genes, such as *RPS19* or *RPL5*. Patient-derived iPSCs recapitulate early hematopoietic defects associated with this disease, including progenitor apoptosis and erythroid maturational arrest [35]. These iPSCs were used as a chemical screening platform, from which SMER28 emerged as a novel candidate therapy for DBA. When used in multiple erythropoiesis models, this compound induced globin gene expression in diseased cells and ameliorated anemia [35].

## Exemplary Uses for iPSC-derived Megakaryocytes and Platelets, and Related Disease Models

Alterations in development and function of megakaryocyte or platelets, which occur in several heritable diseases, can increase bleeding and/or thrombosis risk (Figure 1) [36,37]. Human iPSC-derived megakaryocytes and platelets have been used to study the pathophysiology of platelet disorders, to screen for novel therapies via high throughput platforms, to test for platelet-specific alloantibodies [38], and to test potential cell therapy agents [39].

Human iPSC models have facilitated the study of rare platelet disorders, for which patient samples are very limited. For example, autosomal deletion on chromosome 11q causes Jacobsen syndrome, with an associated platelet defect called Paris-Trousseau syndrome [40,41]. The typical chromosomal abnormality results in deletion of two transcription factors, Friend leukemia virus integration 1 (*FLI1*) and ETS proto-oncogene 1 (*ETS1*). Patient-derived iPSCs and *FLI1*-haploinsufficient iPSCs produced fewer megakaryocyte colonies and fewer megakaryocytes per hematopoietic progenitor cell. In addition, platelets derived from patient-derived or *FLI1*-haploinsufficient cells were quantitatively diminished, had reduced half-lives, and had impaired functional response to thrombin activation [40]. These findings show that *FLI1* deficiency is predominantly responsible for the megakaryocyte and platelet phenotypes associated with Paris-Trousseau Syndrome.

iPSC-derived megakaryocytes have also been used to study mechanisms related to bleeding disorders from dominant negative *Growth Factor Independence 1B* (*GFI1B*) mutations [42]. Patient-derived iPSC hematopoietic differentiation phenocopied the early arrest in megakaryopoiesis seen *in vivo* [42]. In these cells, impaired megakaryocyte development resulted from altered coordinate programming between GFI1B and LSD1 proteins, as DNA binding-incompetent GFI1B protein sequestered and prevented LSD1 from binding DNA [42]. This resulted in deregulated interferon γ (IFNγ) signaling, and altered subsequent expression of key megakaryocyte transcription factors like *MEIS1* [42]. Future work targeting these pathways may lead to novel therapeutic approaches to treat this and related disorders.

Megakaryocytes and platelets from patient-derived iPSCs have also been used to guide gene correction strategies and as novel therapeutic screening reagents. For example, Familial Platelet Disorder (FPD), caused by inherited autosomal dominant *RUNX1* mutations, is characterized by thrombocytopenia, platelet dysfunction, and a predisposition for myeloid malignancies [43]. Cultured patient-derived iPSCs have recapitulated key disease features, including decreased megakaryocyte colony formation and decreased quantities of CD41a^+^CD42b^+^ megakaryocytes in lineage specific liquid culture [43,44]. These defects were normalized following genetic correction. In addition, patient-derived megakaryocytes have been used to identify novel therapeutic targets. Expression analyses showed that *NOTCH4* was upregulated in FPD iPSC-derived megakaryocytes [45]. *NOTCH4* deletion, or inhibition of NOTCH4-related signaling, enhanced megakaryocyte differentiation in diseased cells and even improved megakaryocyte production in cultures from wild type iPSCs [45]. These findings revealed a role for NOTCH signaling in FPD pathophysiology, suggesting that pharmacologically activating NOTCH4 signaling may enhance *in vitro* hematopoiesis and megakaryopoiesis in both FPD and control backgrounds.

## Exemplary Uses for iPSC-derived Myeloid and Lymphoid Cells, and Related Immunodeficiency Syndromes

iPSC-derived myeloid and lymphoid cells have been used to study disease biology, gene correction strategies, and novel precision therapeutics for immunodeficiency syndromes (Figure 1) [46]. For example, iPSC models have clarified genes and developmental defects underlying severe combined immunodeficiency (SCID), a clinically heterogenous syndrome caused by defective T and B cell immunity [47]. Clinical defects can arise from cell-autonomous defects impacting hematopoietic stem and progenitor cell biology, or immune cell differentiation. SCID can also result from abnormalities in bone marrow or thymic tissue, which provide the stromal environment for hematopoiesis and immune cell development. Hematopoietic stem cell transplantation (HSCT) is the only curative therapy for this disorder. Notably, lymphoid cell studies require definitive-like hematopoietic iPSC model systems, as primitive hematopoiesis does not produce this lineage.

Patients with *PAX1* mutations can present with otofaciocervical syndrome type 2 and SCID. Some patients with mutations affecting PAX1 DNA binding and transcriptional regulation activities fail to reconstitute their T cell compartments following otherwise successful HSCT [48]. T cells from these patients were hypofunctional. The use of patient-derived iPSCs showed aberrant differentiation to thymic epithelial progenitor cells, confirming a role for *PAX1* deficiency in altering T cell development via perturbation of thymic epithelial tissues. The mechanism by which patients develop SCID in the context of *PAX1* deficiency is similar to *FOXN1*, *CHD7*, or DiGeorge Syndrome, in that the disease occurs as a consequence of abnormal thymic development.

RAG2-SCID is a severe form of the disease that is associated with few or no T or B cells. This form of SCID is typically associated with immune cell developmental defects. Although murine models have indicated specific defects, the stages affected in human immune cell development were unknown. Themeli et al. generated an iPSC line from RAG2-SCID patient cells and used homologous recombination to create an isogenic control line to examine these cells during T cell differentiation [49]. Both RAG2-SCID and control iPSC-derived cells differentiated normally in the early stages of hematopoiesis, but T cell development was severely impaired at multiple stages of differentiation using the patient-derived line. Specifically, there were profound reductions in CD7^+^CD5^+^ early lymphoid cells, CD4^+^CD8^+^ T cells, and TCR rearrangements. These findings demonstrated that aberrant development in diseased cells begins in early lymphoid differentiation and persists throughout T cell development.

Patient-derived iPSCs have also provided a testing platform for novel gene correction strategies to ameliorate immunodeficiency syndromes. For example, patients with chronic granulomatous disease (CGD) have recurrent bacterial and fungal infections caused by defective phagocytic functions. By correcting one of the NADPH oxidase mutations responsible for 90% of the cases of CGD in a patient-derived iPSC line, NADPH oxidase function and cellular bacterial killing activity were restored *in vitro* [50].

iPSC models can also provide cellular screening platforms for novel immunodeficiency syndrome treatments. For example, interferon γ (IFNγ) defects underlie genetic predisposition to mycobacterial infections [51]. IFNγ is a key macrophage stimulating factor. iPSC-derived macrophages from immunodeficient patients with defective IFNγ responses, caused by deficiency in IFNγR1, IFNγR2, or STAT1, had normal morphology and displayed normal IFNγ-independent phagocytosis. However, these diseased cells were defective in containing weak mycobacterial infection *in vitro*, consistent with the related clinical phenotype [51]. These experiments provide proof-of-principal for iPSC-derived cells to be used as drug and treatment screening platforms, and for mechanistic studies into disorders related to IFNγ pathway deficiency.

## Exemplary Uses for IPSC-derived Blood Cells in Studying Hematopoietic Malignancies

iPSC-derived blood cells have also facilitated studies of hematopoietic malignancies, including leukemias and lymphomas (Figure 1). These *in vitro* systems have helped reveal mechanisms and as screening platforms to identify novel therapies for oncogenic processes. To identify genetic and mechanistic factors driving familial myeloproliferative neoplasms, iPSCs were derived from patients and families with predisposition to autosomal dominant adult-onset myeloid malignancies [52]. These patients frequently had *JAK2*, *MPL*, or *CALR* mutations, which can cause sporadic essential thrombocythemia and a predisposition to acute myeloid leukemia. However, copy number variation (CNV) analysis in these families also identified variations in other genes, such as *ATG2B* and *GSKIP*. Patient-derived iPSCs were differentiated to hematopoietic progenitor cells and those containing the CNV produced ten-fold more colonies than controls, with hypersensitivity to thrombopoietin and erythropoietin [52]. Patient-derived cells formed megakaryocyte colonies in the absence of thrombopoietin, demonstrating cytokine independence. Some patient-derived clones showed erythroid or megakaryocyte lineage biases that were related to concurrent *JAK2* and/or *TET2* mutations. Gene expression analyses indicated that *ATG2B* and *GSKIP* were overexpressed in hematopoietic cells containing the CNV, suggesting that these genes might be responsible for the *in vitro* and clinical phenotype. Indeed, lentiviral transduction of *ATG2B*- and *GSKIP*-targeted shRNAs in diseased iPSC-derived megakaryocyte progenitors reversed thrombopoietin-independent megakaryocyte colony formation. Knockdown of these genes also inhibited megakaryocyte colony formation in cultured CD34^+^ hematopoietic stem cells. Hence, the CNVs identified in these families increased hematopoietic progenitor cell production and cytokine hypersensitivity via overexpression of *ATG2B* and *GSKIP*, explaining the familial predisposition to myeloid malignancies.

Patient-derived iPSCs have also helped elucidate the mechanisms by which Trisomy 21 (T21) and related *GATA1* mutations impact hematopoietic development. Individuals with T21 often have polycythemia and thrombocytopenia, and as infants, are predisposed to a preleukemic transient myeloproliferative disease (TMD) associated with *GATA1* mutations. While TMD has fetal liver origins due to dysregulated hematopoiesis, subsequent acute megakaryoblastic leukemia (AMKL) evolves through acquisition of additional mutations, often affecting core cohesion components. Patient-derived T21 iPSC models with wild type *GATA1* and mutant *GATA1* recapitulated key features of their disordered hematopoiesis, including enhanced erythropoiesis or megakaryopoiesis, respectively [53–55]. The iPSCs reprogrammed from infants with TMD and associated with exclusive expression of the truncated GATA1 ‘short’ transcriptional variant showed accelerated hematopoietic progenitor production through RUNX1/ETS2/ERG signaling, resulting in aberrant megakaryopoiesis and phenocopying transient myeloproliferative disorder [56]. Indeed, the ‘full-length’ isoform of GATA1 is critical for normal hematopoiesis, particularly in the erythroid lineage [55].

iPSC models have also served as a screening platform for chemotherapeutic strategies. Juvenile myelomonocytic leukemia (JMML) is a rare myeloproliferative neoplasm that affects young children and remains associated with high morbidity and mortality. JMML is often caused by mutation in Ras pathway genes, resulting in hyperactive Ras/MAPK signaling in myeloid cells [57,58]. iPSCs derived from patients with *PTPN11* or *CBL* mutations recapitulated *in vivo* disease phenotypes, including an increased proliferative capacity in myeloid cells, with constitutive activation and/or hypersensitivity to granulocyte macrophage colony-stimulating factor [57]. These cells have been used as a screening platform for targeted kinase inhibitor therapies, revealing heightened sensitivity to mTOR inhibition, of *PTPN11*-mutated JMML to MEK inhibition, and *CBL*-mutated JMML to JAK inhibition [58].

Chimeric antigen receptor T (CAR-T) cell therapy, a significant and promising breakthrough in cancer treatment [59], has the potential to be enhanced by using iPSC-derived products. Current CAR-T strategies rely on manipulation and expansion of individual patient-derived primary cells. Future alternatives to expand applicability, reliability, and efficacy of this approach could utilize pre-cultured cells as immunotherapeutic agents ‘off the shelf’ [60]. In a proof of principle study, Li et al. identified CARs targeting the tumor antigen mesothelin [60]. These CARs were optimized for activity in natural killer (NK) cells. iPSCs that had been transduced with a construct to express these CARs showed efficient differentiation into NK cells. Engineered cells also demonstrated normal NK activation and effective antigen-specific cytotoxic activity upon contact with mesothelin-expressing K562 cells or A1847 ovarian cancer cells. Further, treatment with CAR-expressing NK cells limited tumor growth and prolonged survival in a murine A1847 ovarian cancer cell xenograft model [60].

## Hematopoietic Contributions to Neurodegenerative Disease

Hematopoietic cells that impact organogenesis and pathophysiology throughout the body include tissue-resident macrophages [16]. The importance of these cells to organ (patho)physiology may be one reason that blood trait-related genes influence a myriad of complex disease states [20]. For example, yolk sac-derived monocytes migrate to the brain and differentiate into resident microglia, which thereafter comprise a lifelong self-sustained cell population (Figure 1) [61]. Microglia are responsible for many of aspects of central nervous system homeostasis, including neuronal synapse pruning. Microglia have been recently recognized to play important roles in neuropsychiatric and neurodegenerative diseases [61,62].

The iPSC technology may be particularly important in modeling neurodegenerative disease, given difficulty in accessing patient tissue and given key differences between human microglia and those in animal models [63]. Several protocols exist to differentiate iPSCs into microglia, with ramifications for cellular remyelinating therapy [64] and microglia-associated disease modeling [61]. For example, gene-edited iPSCs recently demonstrated that *Triggering receptor expression on myeloid cells 2* (*TREM2*) and *Phospholipase C γ2* (*PLCG2*) participate in a common pathway to mediate microglial cell survival, phagocytosis, and lipid metabolism [63]. Loss of either of these genes caused dysfunctional microglial properties that likely underlie an associated increase in propensity for individuals harboring mutations in these genes to develop neurodegenerative disorders, such as Alzheimer’s disease.

## Summary and Future Directions

Patient-derived or gene-edited iPSCs, and derived blood cells, are promising tools for studying genetic mechanisms underlying blood cell related pathology. This commentary has used selected vignettes to show how human iPSC model systems can be used to study mechanisms by which blood cells contribute to hematopoietic and non-hematopoietic disorders, and guide platform development for therapeutic approaches. The refinement of iPSC systems to produce definitive-like hematopoiesis models [23,24] and increase *in vitro* cell production [8,65], will be essential for improving mechanistic studies, small molecule screening, and gene editing approaches. These models may also better support translational research goals in blood bank screening and cellular therapeutics [25]. Future applications of iPSC technology may also dovetail with other model systems, such as *in vitro* organoid models [66] or other platforms [67–69], to reveal complex mechanisms and translational opportunities related to the impact of blood cells on various disease states in nonhematopoietic systems.
